# Frost-free zone on leaves revisited

**DOI:** 10.1073/pnas.2407062121

**Published:** 2024-06-20

**Authors:** Annika Einbock, Franz Conen

**Affiliations:** ^a^Department of Environmental Sciences, University of Basel, Basel 4056, Switzerland

**Keywords:** frost pattern, ice nucleation, epiphytic microorganisms

## Abstract

Particular frost patterns on natural leaves had prompted Yao et al. [Y. Yao *et al*., *Proc. Natl. Acad. Sci. U.S.A.* **117**, 6323–6329 (2020)] to investigate the underlying physics. Their work revealed why on corrugated surfaces ice forms on crests and dries out adjacent grooves. In the absence of frost, in contrast, grooves tend to constitute niches on a leaf where microorganisms are less limited by moisture than in other locations. Here, we show that microorganisms able to nucleate ice before it forms on crests can modify the frosting pattern to their advantage. This ability might drive in cold arid environments the association between certain microorganisms and plants.

The morning after a freezing cold night hoar frost can be observed on leaves and other structures. On the upper side of corrugated leaves, ice crystals mostly grow on elevated parts, such as crests between veins, whereas grooves along veins often remain ice-free. The observation of such patterns had inspired Yao et al. (1) to investigate the related frosting process more closely on similar, engineered surfaces. Their experiments and modeling show a smaller water vapor flux into grooves than toward crests. Consequently, dew droplet size increases from groove to crest, where ice formation starts because the likelihood of freezing increases with the size of droplets. Ice on a crest lowers the water vapor pressure and leads to the evaporation of smaller droplets in adjacent grooves. Under warmer conditions, in contrast, grooves along veins constitute protected niches where moisture is often less limiting to microbial life than elsewhere (2, 3). Such differences in microhabitat make locations in grooves more frequently colonized by bacteria than locations on crests (4).

Here, we put forward the idea that microbial ice nucleation has the potential to modify the frosting process as described by Yao et al. (1). The first steps, a greater water vapor flux to crests and a diminishing droplet size toward the bottom of a groove, are unlikely to be affected by the kind of microorganisms inhabiting a groove (Fig. 1 *A* and *B*). Yet, microbial ability to nucleate ice in a groove before this happens on the crest should indeed modify the process (Fig. 1 *B* and *C*). Following the logic of Yao et al. (1), ice formed by microorganisms on the bottom of a groove, while crests bare of such microorganisms are still covered with liquid droplets, should lower the water vapor pressure within the groove and allow the ice crystals there to grow until frost has spread to adjacent crests. By then, water vapor pressure is already as low within the groove as it has just become on adjacent icy crests, protecting the groove from drying out (Fig. 1*C*). At daybreak, when leaf temperature rises above 0 °C and water becomes available to microorganisms in this groove, other grooves, occupied by microorganisms unable to nucleate ice, would remain dry when ice melting on crests is either retained by trichomes (5) or evaporates on site. However, only a small fraction of bacterial cells on a leaf is able to nucleate ice (6). Without such cells, leaves freeze at around −8 °C (7). The first bacterium discovered to nucleate ice little below 0 °C was *Pseudomonas syringae* (8).

**Fig. 1.**
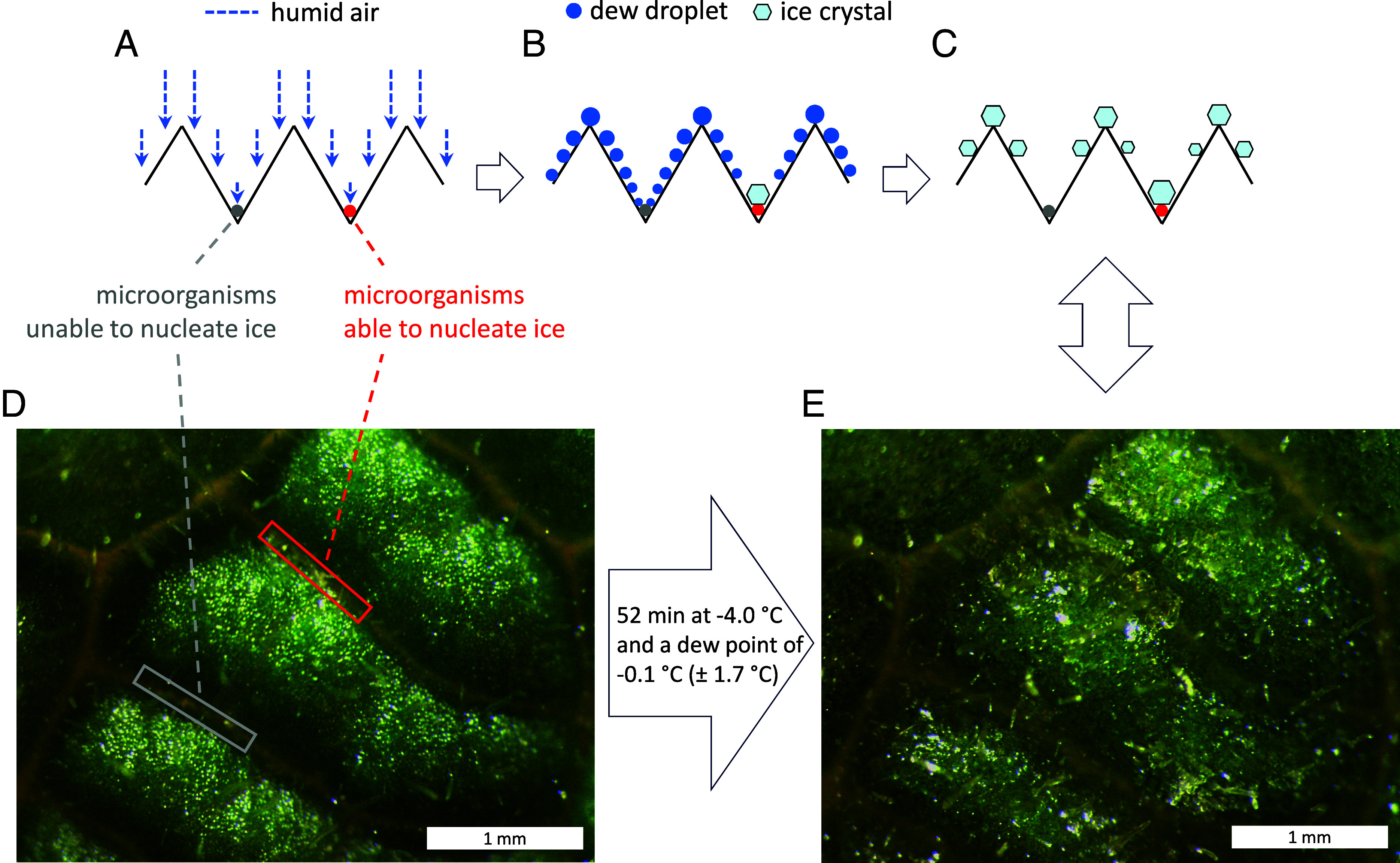
Schematic illustration of the frosting process on corrugated surfaces, adapted from Yao et al. (1) and modified by adding microorganisms to grooves. (*A*) Condensation of water vapor onto the cold surface. (*B*) Larger droplets have formed on crests and smaller ones in grooves. Unlike in Yao et al. (1), first ice does not form on crests, but in the groove occupied by microorganisms able to nucleate ice. (*C*) The frost pattern in the left-hand part of the illustration is the same as in Yao et al. (1), whereas on the right-hand side, microorganisms have facilitated the growth of an ice crystal, which upon melting will provide them moisture. (*D*) Photograph of a leaf section of *D. purpurea* mounted on a cold plate at the beginning of the experiment. The locations where microorganisms were applied are framed. (*E*) Same view as (*D*), but 52 min later when the frost pattern schematically shown in (*C*) has formed on the leaf.

We performed experiments with leaves of *Digitalis purpurea*, a biennial flower native of Western Europe and North Africa, where it is frequently found in woodland clearings or other disturbed sites. Leaves have a millimeter-size structured surface covered with small trichomes. Grooves were furnished either with microorganisms able or unable to nucleate ice, and frost formation was observed at −4.0 °C for about 1 h. Humid air depositing on the cooled leaf surface rendered the fine powdery trace of applied microorganisms quickly invisible. Initial ice crystals appeared within 5 to 10 min exclusively in the position where microorganisms able to nucleate ice had been applied, but not in other groove positions. After 15 to 20 min, ice had also formed on crest positions. By 52 min into the experiment, most parts of the leaf area clearly exhibited the frost pattern described by Yao et al. (1), except for a rich ice deposit in the groove section furnished with microorganisms able to nucleate ice (Fig. 1*E*). If ice had not formed there before it did so on adjacent crests, this section of the groove would also have desiccated during the frosting process. Therefore, it is only by initiating the first ice nucleation on a leaf that microorganisms in a groove can modify a frosting process that would otherwise give them a dry start into the day after a freezing cold night. The presence of such microorganisms and their spatial distribution on natural leaves could be tested in freezing assays with leaf imprints on a flat cold plate coated with a thin layer suitable to retain microorganisms. Their effect on ice nucleation could then be studied in isolation from other leaf properties possibly affecting the freezing pattern.

Spatial distribution and composition of bacterial communities on leaves are related to leaf microtopography (9). Bacteria on leaf surfaces are often confronted with water scarcity, which substantially affects their survival and growth (10). Bacteria have developed multiple strategies to reduce the risk of fatal desiccation, including the production of biosurfactants (11), aggregation (12), and potentially the colonialization of wetter leaf niches (2, 4). Modifying the frosting process as shown here constitutes a so far unexplored mechanism by which epiphytic microorganisms might further alleviate water limitation. It acts at times when temperature fluctuates around 0 °C and probably facilitates the association between host plants with structured surfaces and microorganisms able to nucleate ice little below 0 °C. Equally benefitting from the accumulation of ice in grooves would be microorganisms that are themselves unable to nucleate ice but are able to live together with those who do. A hint that plants with specific surface structures filter for such microbial communities is found in the hyperarid Hexi-Corridor of NW China, where temperature (in °C) changes sign on around 150 d a year. Among the three shrub species investigated by Liu et al. (12), only *Caroxylon passerinum* has grooves, between densely packed, small, thick, and trichome-covered leaves. These leaves host a much larger fraction of *Pseudomonads* in a more interconnected network of bacterial communities than observed on the smooth leaves without trichomes of the two other shrub species (13). Another clue is found in Hill et al. (14) who identified on leaves of *Cercocarpus montana “an unexpectedly high population”* of bacteria able to nucleate ice. *C. montana* is native to the semiarid climate of the Laramie Mountains (USA). Its leaves feature both, deep pinnate venation and trichomes. As in the Hexi-Corridor, the ability to nucleate ice in leaf veins might reduce water limitation of epiphytic microorganisms in the Laramie Mountains, where temperatures (at Laramie) fluctuate around 0 °C on about 130 d per year.

It would be interesting to see the frost pattern of these shrubs with differing surface structure in situ after a cold night. While the general frost pattern observed on leaves is governed by physical processes interacting with leaf surface structure, deviations from the expected pattern (Fig. 1*D*) could indicate the presence of ice-nucleating microorganisms and guide more detailed investigations of the association between leaves and microorganisms under harsh conditions.

## Materials and Methods

We conducted several runs with the same kind of result as shown in the photographs in Fig. 1. For each experimental run, a small piece (ca. 5 mm x 5 mm) was excised from a young leaf of *D. purpurea* and placed on a cold plate (15) covered with a thin layer of Vaseline (Verfora, Switzerland) to ensure good thermal contact. With a micromanipulator (MK1, Singer Instruments, United Kingdom), we applied tiny amounts of dry microbial cells to grooves. One groove was always furnished with *P. syringae* (Snomax®, USA), able to nucleate ice, and another groove with *Saccharomyces cerevisiae* (Mondamin, Germany), unable to nucleate ice (Fig. 1*D*). The freezing temperature of Snomax® had been determined on the same cold plate without a leaf sample. Starting at 0 °C, droplets (2 µL) of pure water with Snomax® were cooled in 0.5 °C steps. They froze reliably at −4.0 °C within less than a minute, whereas droplets without Snomax® did not freeze above −12.0 °C. Freezing of particles with ice nucleation active sites is initiated when the site-specific temperature is reached, with little time dependence (16).

The prepared cold plate with the leaf sample was transferred to a thermostat cabinet (10 °C, relative humidity 50%), where the leaf surface was illuminated with a spotlight from a shallow angle and observed from above with a stereo-microscope (M5, Wild, Switzerland) at 25x magnification and an eye-piece camera (AM4025X, Dino-Lite, The Netherlands) fitted to one of its ocular tubes. Once temperature and relative humidity had stabilized inside the cabinet, the experiment was started by setting the cold plate to −4.0 °C and recording images.

The number of days with temperature (in °C) changing sign was derived from daily minimum and maximum temperature at the meteorological station Guazhou, 75 km NW of the field site described by Liu et al. (13) and the meteorological station Laramie, 10 km SW of the *C. montana* sampling location in Hill et al. (14) (data source: https://meteostat.net).

## Data Availability

All study data are included in the main text.
